# Local treatment of burns by honey is not appropriate

**DOI:** 10.11604/pamj.2016.23.147.8707

**Published:** 2016-03-31

**Authors:** Hassen Ben Ghezala, Najla Feriani

**Affiliations:** 1 Service Universitaire des Urgences et de Réanimation Médicale; 2 Hôpital régional de Zaghouan, Faculté de médecine de Tunis, Tunisie; 3 Service Universitaire de Chirurgie Générale

**Keywords:** Burns, local treatment, honey

## Image in medicine

According to the world health organization (WHO), management of burns should be well codified and organized such as in trauma patients. The severity of burns is determined usually by the burned surface area, the depth of burn and other considerations. Morbidity and mortality rises with increasing burned surface. Initial local treatment of burn lesions should be focused on speedy healing and prevention of infection. In our African countries and in Tunisia, we still have some strange traditional local treatment of burns. In this rare case report, a seventeen years old young teenager which had a burned leg and back of foot was treated by local administration of honey. After a week of treatment, he was admitted to the emergency department of the regional hospital of Zaghouan in Tunisia. At examination, we found a second degree burns with purple cutaneous blisters covered with white adhesions. We found also sections of dying skin. This inappropriate initial local treatment with honey application could lead to severe complications. After gentle debridement with 0.25% chlorhexidine solution, and gentle scrubbing, all necrotic tissue was removed. After daily change of dressing and systemic antibiotics, the lesions recovered totally. The teenage was discharged from hospital after five days.

**Figure 1:**
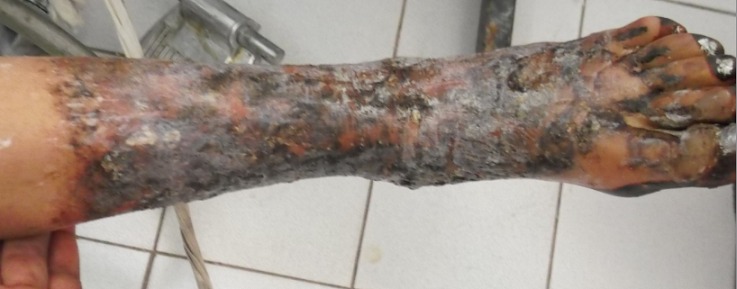
Second degree burns two days after local treatment by honey

